# Villaret's Syndrome Secondary to Internal Carotid Artery Dissection: A Case Report

**DOI:** 10.7759/cureus.114678

**Published:** 2026-08-17

**Authors:** Niazi Khairi, Karishma Shamarukh, Mahantesh Kuppasad, Robert Barker

**Affiliations:** 1 Department of Internal Medicine, Frimley Park Hospital, Camberley, GBR; 2 Department of Stroke Medicine, Frimley Park Hospital, Camberley, GBR; 3 Department of Radiology, Frimley Park Hospital, Camberley, GBR

**Keywords:** carotid dissection, horner’s syndrome, internal carotid artery dissection, multiple cranial nerve palsies, villaret's syndrome, villaret syndrome

## Abstract

Villaret’s syndrome is a rare neurological condition characterized by ipsilateral palsies of the cranial nerves (CNs) IX, X, XI, and XII in association with Horner’s syndrome. We report a 52-year-old man presenting with headache, hoarseness of voice, and dysphagia following strenuous exercise. Physical examination revealed left-sided lower CN palsies and partial Horner’s syndrome, implying pathology in the retroparotid space, the only anatomical site where these nerves are colocated. Initial stroke computed tomography head and angiogram were reported as unremarkable, but a subsequent magnetic resonance imaging (MRI) of the head and neck demonstrated an expansile cervical internal carotid artery dissection (ICAD) as the underlying cause. The patient was managed with dual antiplatelet therapy and achieved full symptomatic resolution within six weeks. This case underscores that ICAD can present as Villaret’s syndrome without cerebral ischemia. It also highlights that a "normal" computed tomography angiography does not exclude dissection; targeted MRI is more sensitive for detecting intramural pathology in suspected lower CN syndromes.

## Introduction

Villaret’s syndrome, or posterior retropharyngeal syndrome, is defined by ipsilateral palsies of the lower four cranial nerves (CNs), the glossopharyngeal (IX), vagus (X), accessory (XI), and hypoglossal (XII) in association with Horner’s syndrome (ptosis, miosis, anhidrosis, and apparent enophthalmos), reflecting pathology in the retroparotid space, a narrow anatomical corridor where these nerves course alongside the cervical sympathetic chain [1,2]. Villaret’s syndrome is often confused with Collet-Sicard syndrome, in which the same CNs are involved, but the sympathetic function is spared [3].

Clinical manifestations typically include dysphagia and loss of taste (IX), dysphonia and palatal deviation (X), weakness in shoulder shrugging and head rotation (XI), tongue hemiatrophy or deviation (XII), and palpebral ptosis, miosis, and facial sweating disturbances (Horner’s syndrome due to sympathetic chain involvement) [4].

While historically associated with penetrating trauma [1], modern cases are often secondary to neoplasms [5-8] or vascular events such as internal carotid artery dissection (ICAD) [9,10]. Management of ICAD-induced Villaret’s syndrome typically centers on antithrombotic therapy, though surgical or endovascular interventions may be pursued if medical management is insufficient [11]. Because these presentations can be misinterpreted or coexist with an acute stroke, a high index of clinical suspicion is required to initiate appropriate investigations and timely treatment to reduce the risk of embolic ischemic complications. Our case highlights the clinical trajectory of a 52-year-old man presenting with exercise-induced Villaret’s syndrome secondary to ICAD, which was successfully managed with medical therapy, leading to a full symptomatic recovery.

## Case presentation

A 52-year-old Caucasian man with no significant past medical history presented to the emergency department with a one-week history of gradually progressive frontal headache, hoarseness of voice, and dysphagia, following a session of strenuous physical exercise.

**Figure 1:**
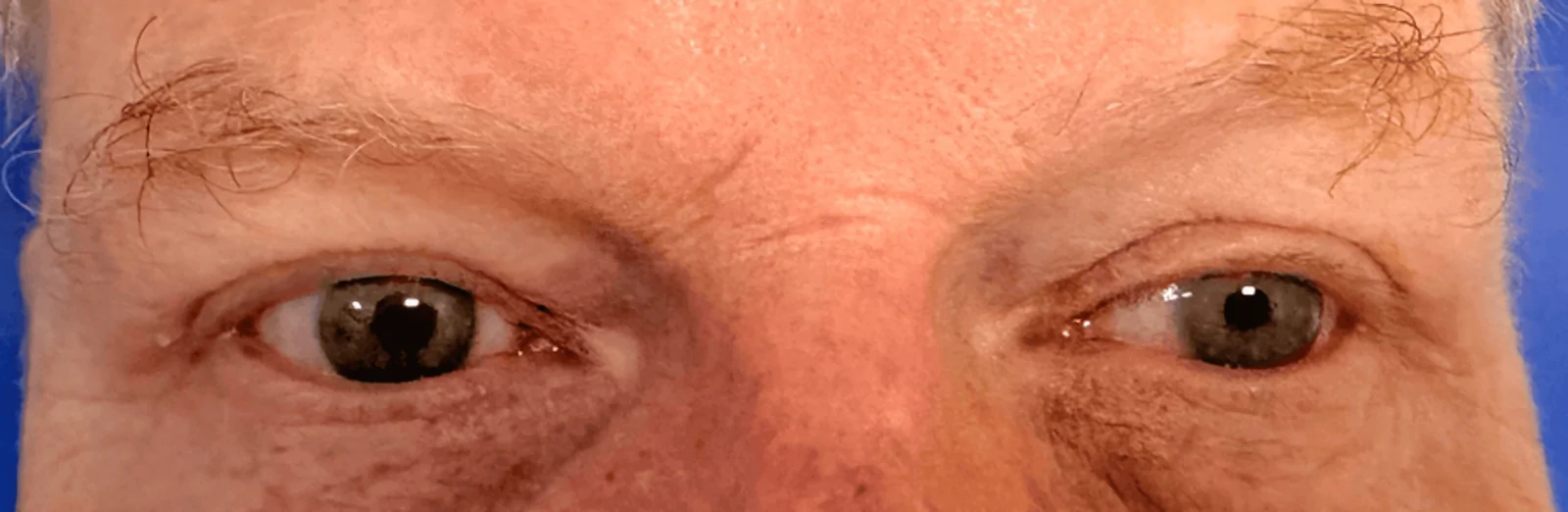
Initial bedside photography demonstrating acute left-sided miosis and mild ptosis, representing dynamic evolution of acute left-sided oculosympathetic paresis (Horner's syndrome)

**Figure 2:**
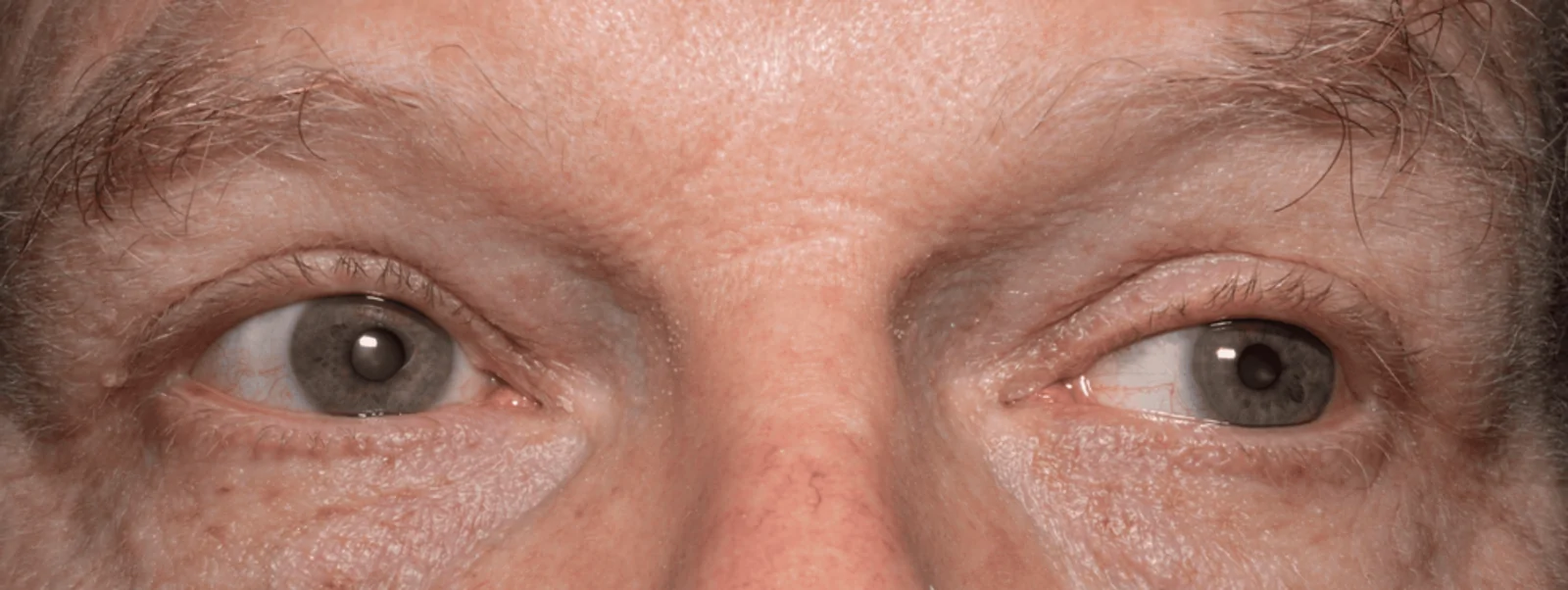
Official clinical photography obtained on day 2, showing spontaneous resolution of the miosis with persistent left-sided ptosis

On initial assessment, his National Institutes of Health Stroke Scale score was 1, reflecting mild dysarthria [12]. Physical examination revealed a partial left-sided Horner’s syndrome, characterized by miosis and mild ptosis (Figure 1). Interestingly, an examination the following day revealed a dynamic clinical picture, in which the acute left miosis had resolved while the left-sided ptosis persisted (Figure 2). There was no associated upper or lower limb weakness, and cerebellar examination was normal.

**Figure 3:**
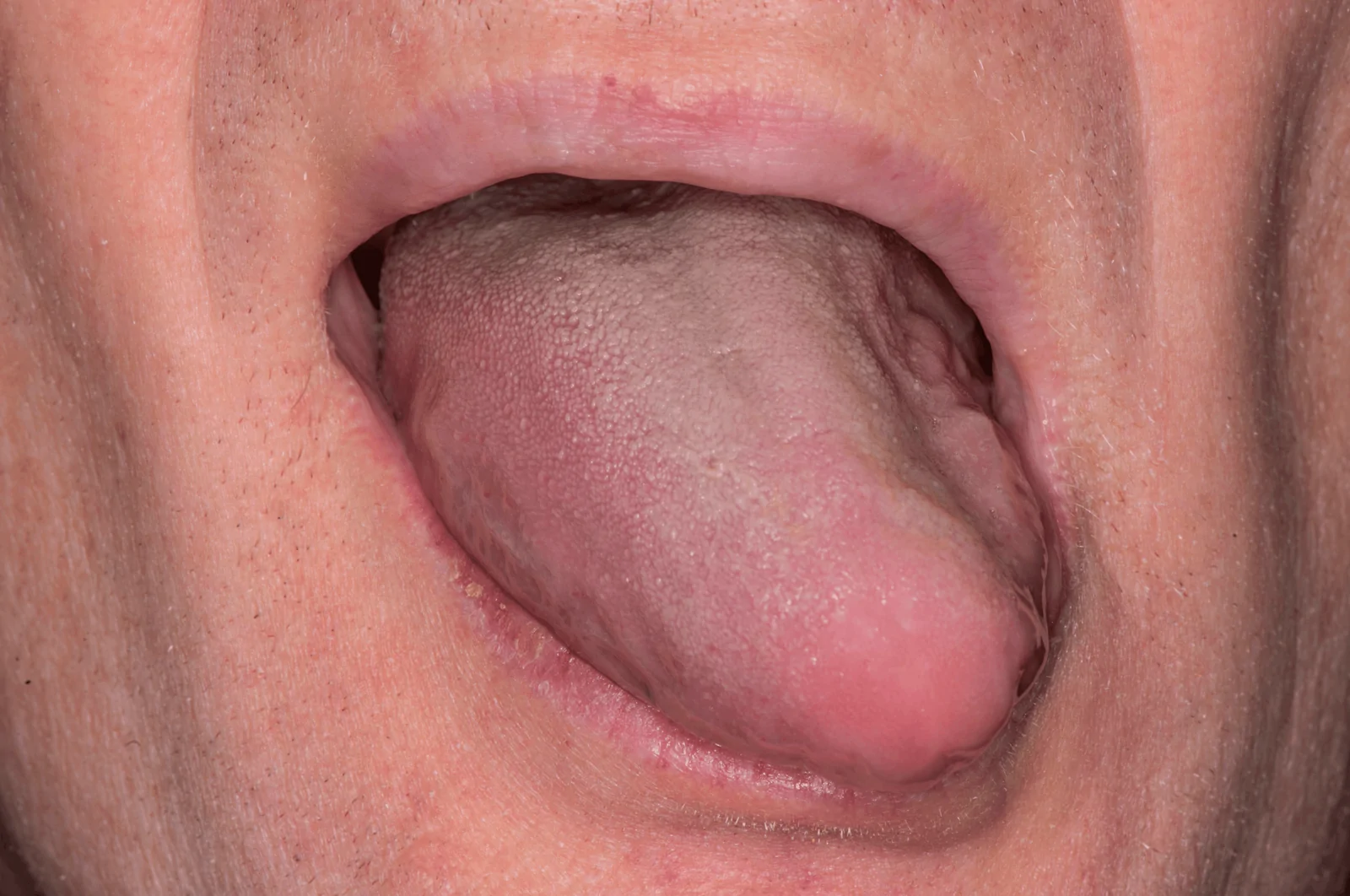
Clinical photography demonstrating marked deviation of the tongue to the left upon protrusion, accompanied by localized fasciculations, representing lower cranial nerve XII palsy

**Figure 4:**
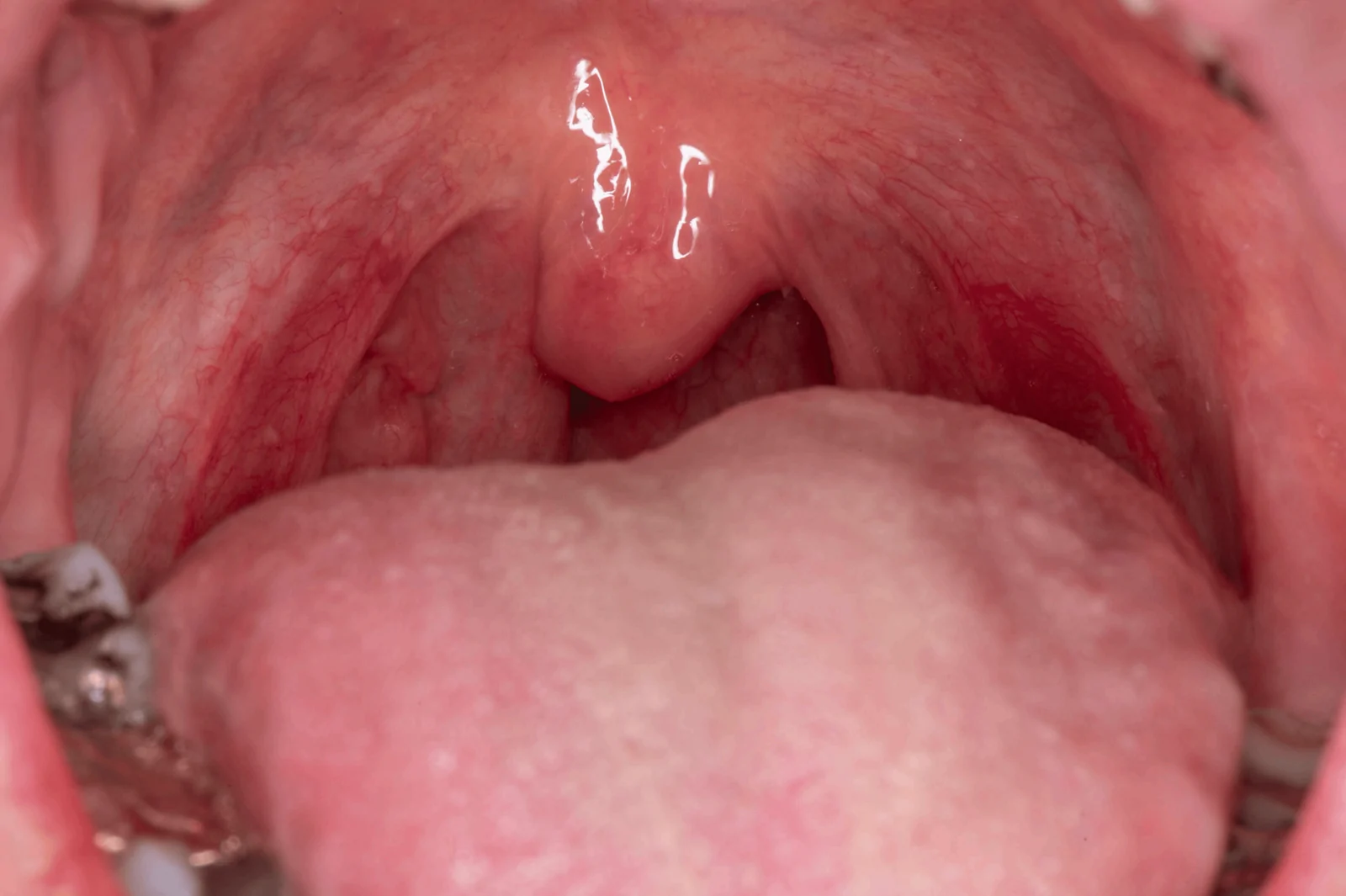
Intraoral clinical photography demonstrating asymmetric palatal elevation and contralateral deviation of the uvula to the right, representing cranial nerve X palsy

The neurological assessment further revealed multiple ipsilateral lower CN palsies. These were characterized by leftward deviation of the tongue upon protrusion with associated fasciculations (CN XII palsy) (Figure 3), reduced left-sided palatal elevation and contralateral deviation of the uvula to the right (CN X palsy) (Figure 4), a diminished left gag reflex (CN IX palsy), and mild weakness of the left shoulder shrug (CN XI palsy). Together, this clinical constellation of an oculosympathetic paresis combined with CN IX, X, XI, and XII palsies was diagnostic of Villaret’s syndrome.

**Figure 5:**
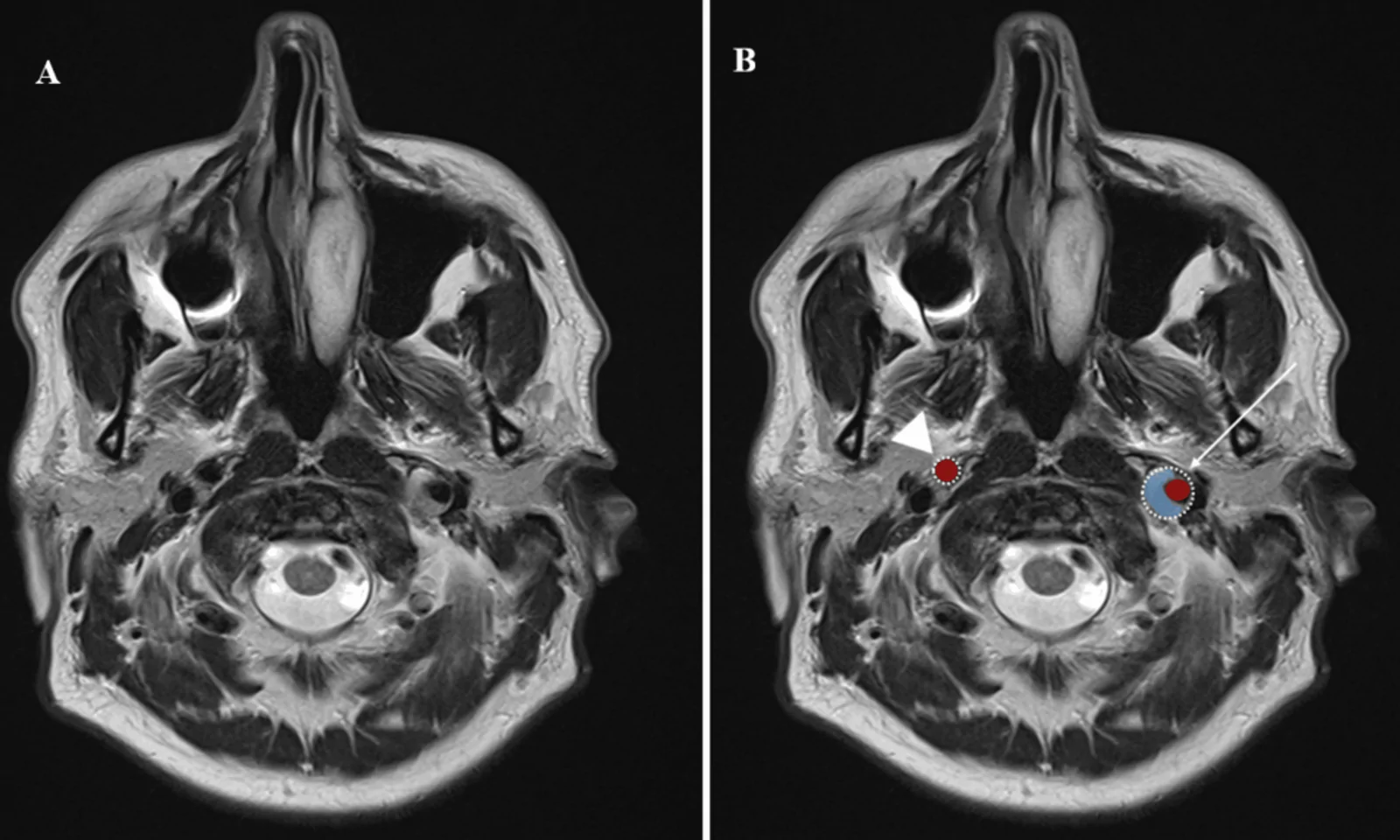
Axial T2-weighted MRI (A) and annotated image (B) at a level below the skull base showing the distal right (arrowhead) and left (arrow) internal carotid arteries. The left ICA diameter is expanded (dotted line) due to an intramural hematoma (blue area), which is easily identified by the signal contrast between the T2-hyperintense hematoma and the T2-hypointense flowing blood within the arterial lumen ICA: internal carotid artery; MRI: magnetic resonance imaging

**Figure 6:**
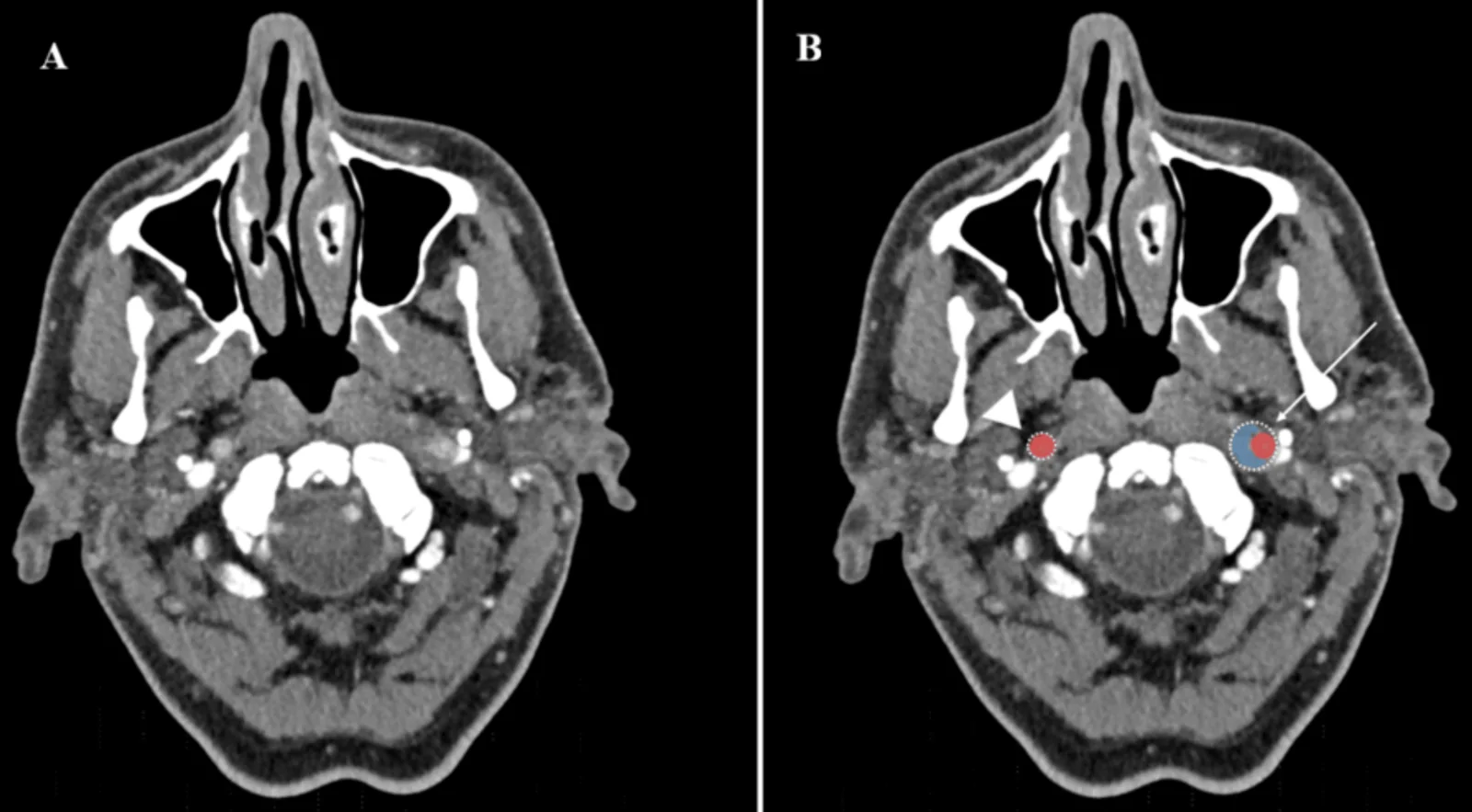
Axial CTA (A) and annotated image (B) at a level below the skull base showing the distal right (arrowhead) and left (arrow) internal carotid arteries. The left ICA (dotted line) is expanded due to the presence of an intramural hematoma (blue area); however, only minimal luminal stenosis is observed (red circle) CTA: computed tomography angiography; ICA: internal carotid artery

**Figure 7:**
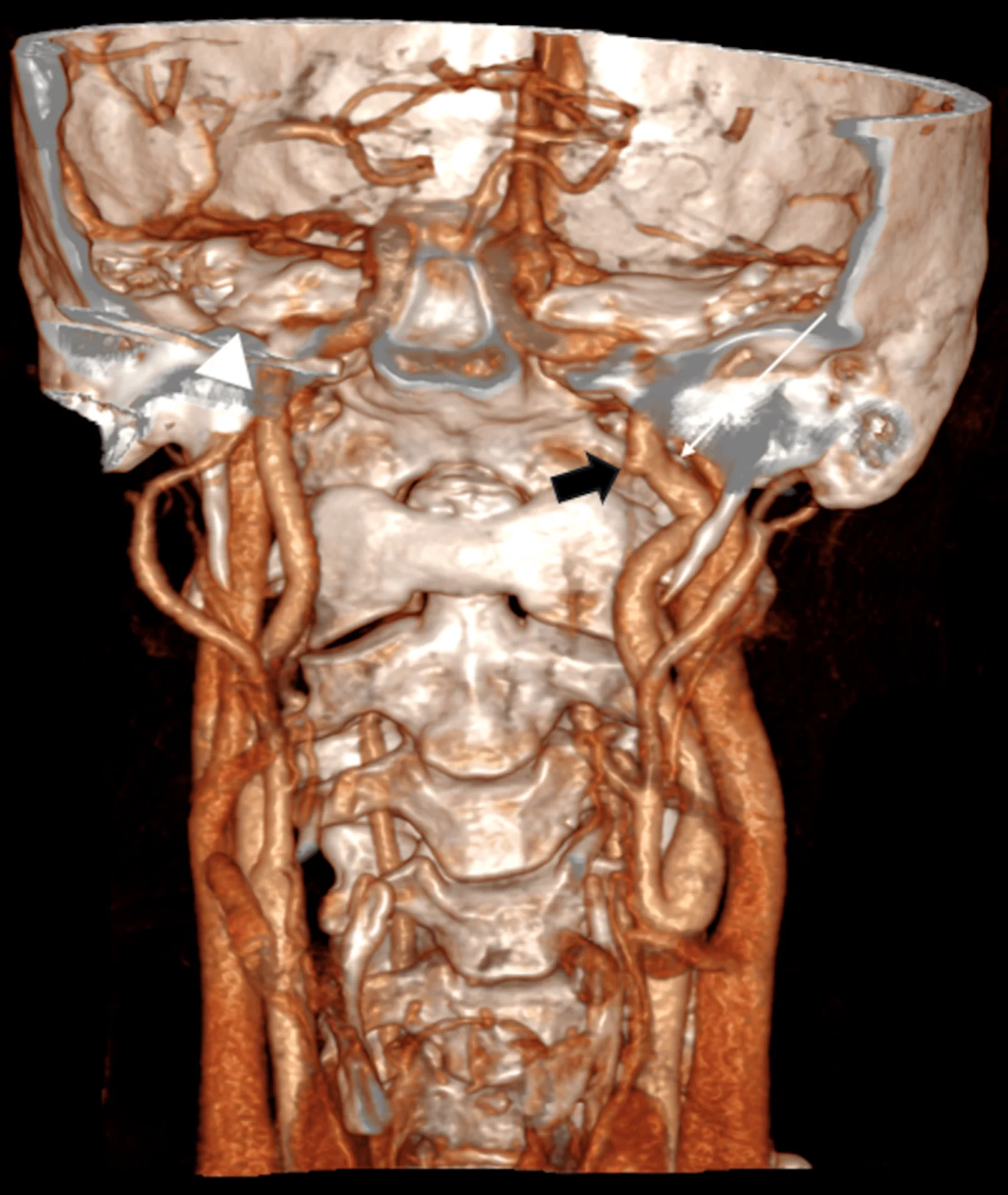
Surface-shaded CTA reconstruction demonstrating symmetric luminal diameter of the right (arrowhead) and left (white arrow) internal carotid arteries, with subtle vessel wall irregularity on the left secondary to dissection (black arrow) CTA: computed tomography angiography

Initial vital signs were significant for hypertension (167/100 mmHg); however, heart rate (71 bpm) and respiratory rate (18 bpm) were stable, and an electrocardiogram showed sinus rhythm. An acute computed tomography (CT) brain and computed tomography angiography (CTA) for suspected stroke demonstrated no acute ischemic changes or macrovascular occlusion. A subsequent magnetic resonance imaging (MRI) of the head and neck 24 hours after admission revealed an expansile, crescent-shaped intramural hematoma within the cervical segment of the left internal carotid artery (ICA), confirming an acute carotid artery dissection causing minimal stenosis of the arterial lumen (estimated at <20% on North American Symptomatic Carotid Endarterectomy Trial-style assessment) (Figure 5) [13]. Brain and spine imaging, including diffusion-weighted imaging (DWI), demonstrated normal brain and brainstem parenchyma with no evidence of acute ischemia, and a normal upper cervical cord with no features of compression or signal abnormality. A retrospective review of the initial CTA confirmed that the dissection was subtle and difficult to identify prospectively due to the minimal luminal stenosis present (Figures 6, 7).

Laboratory investigations, including a full blood count, kidney and liver function tests, and a coagulation profile, were normal. His lipid panel was unremarkable apart from an elevated non-high-density lipoprotein cholesterol level of 4.7 mmol/L (reference range: <4.0 mmol/L). A vasculitis workup, including serum homocysteine levels and inflammatory markers, namely C-reactive protein and erythrocyte sedimentation rate, was unremarkable.

A formal speech and language therapy (SALT) assessment identified mild oropharyngeal dysphagia with reduced oral control and evidence of airway penetration, along with nasal speech. The patient was commenced on dual antiplatelet therapy (DAPT) with aspirin 75 mg and clopidogrel 75 mg, once daily [14,15]. High-intensity statin therapy (atorvastatin 80 mg) for dyslipidemia and perindopril 2 mg for blood pressure optimization were also initiated. Strict swallowing precautions were implemented, including International Dysphagia Diet Standardisation Initiative Level 1 (Slightly Thickened) fluids, and he was referred to the community SALT team for ongoing rehabilitation [16].

At follow-up six weeks later, the patient's presenting symptoms had completely resolved. He was discharged from the community SALT service, and his blood pressure remained well-controlled within the target range. The DAPT regimen was continued for a total duration of three months, to be followed by long-term clopidogrel monotherapy. The patient was subsequently discharged to the care of his general practitioner.

## Discussion

This case is particularly noteworthy because it illustrates how ICAD can present as a complete Villaret’s syndrome while remaining "occult" on initial CTA. While CTA is the standard of care for many vascular emergencies and acute stroke imaging, its primary focus on luminal patency can lead to a "false negative" when a dissection results in outward expansion of the arterial wall with preserved luminal flow, and consequently, initial CTA may be reported as unremarkable. This highlights a critical diagnostic pitfall: a "normal" CTA does not exclude all vascular pathology, particularly when obtained and interpreted in an acute stroke call, and when lower CN palsies are present.

While first described by Villaret in 1916 following battlefield trauma [1], modern cases stem from diverse neoplastic, infectious, and vascular causes. Regardless of etiology, the anatomical mechanism remains constant: neural compression within the narrow retroparotid space. Neoplastic causes include primary tumors (cervical paragangliomas [5], schwannomas [6]) and nodal metastases from prostate [7] and lung [8] malignancies. Nonneoplastic causes range from skull base osteomyelitis [17] and jugular vein thrombosis [9] to ICAD [10], as seen in our patient.

To accurately diagnose this presentation, Villaret’s syndrome must be differentiated from other closely related skull-base neuropathy syndromes, such as Vernet (involving CN IX, X, and XI) and Collet-Sicard (CN IX, X, XI, and XII) syndromes, by identifying the exact combination of involved structures [3]. Villaret’s syndrome is primarily distinguished by sympathetic nerve involvement (manifesting as Horner syndrome) due to a mass effect in the retroparotid space, where the sympathetic fibers are in close anatomical proximity to the carotid sheath. In our patient, an outward mural expansion from the ICAD simultaneously compressed all adjacent pathways, producing this classic clinical constellation.

ICAD classically presents with ipsilateral headache or neck pain, partial Horner’s syndrome (miosis and ptosis), and features of distal embolic cerebral or retinal ischemia [18]. CN palsies are a rare complication of ICAD, with two primary pathophysiological theories for the mechanism: first, mechanical compression where an intramural hematoma or pseudoaneurysm exerts local pressure on the lower CNs due to their close anatomical proximity to the carotid sheath [18,19], and second, an ischemic mechanism involving the interruption of the vasa nervorum (the nutrient vessels supplying the nerves). CN palsy as the sole manifestation of ICAD is exceedingly rare [19], and the complete constellation of Villaret’s syndrome in the absence of intracranial ischemia is even rarer [10].

Our patient's presentation and imaging findings of a significant intramural hematoma strongly support the mechanical compression hypothesis. This also explains the dynamic evolution of the Horner's syndrome, which presented as transient anisocoria; rather than a complete disruption, the mural hematoma likely exerted partial, localized pressure on the adventitial sympathetic plexus, fluctuating during early hematoma remodeling. The differential recovery, with miosis resolving ahead of ptosis, likely reflects varying mechanical susceptibility of oculosympathetic fibers to compressive injury as the hematoma remodeled and decompressed.

While non-contrast CT is insufficient for diagnosis, helical CTA serves as a rapid screening tool for intimal flaps and stenotic lumens in ICAD [20]. MRI, however, offers superior sensitivity and specificity by visualizing the pathological hallmark of dissection: mural expansion by intramural hematoma. In particular, MRI excels at detecting subtle vessel wall expansion associated with nonstenosing dissections. Subacute hematomas typically demonstrate high signal intensity on T1- and T2-weighted MRI due to the presence of methemoglobin, while paramagnetic properties cause susceptibility artifact that may occasionally mimic diffusion restriction. The degree of luminal narrowing caused by the intramural hematoma remains variable [21].

Once a diagnosis of ICAD is confirmed, the primary therapeutic goal is to prevent thromboembolic complications secondary to intimal disruption using antithrombotic therapy (antiplatelets or anticoagulation) [14,15]. Where medical management is insufficient, surgical or endovascular interventions may be pursued. Open surgical options involve direct access to the vessel for primary repair, resection, or arterial bypass, whereas endovascular approaches employ minimally invasive techniques such as angioplasty and stenting [11]. Our patient’s clinical course followed this conventional hierarchy; initiating DAPT was sufficient to manage the dissection without necessitating second-line surgical or endovascular rescue.

The overall mortality rate of carotid artery dissection remains low (<5%) [22], and the long-term prognosis for survivors is highly favorable, with approximately 94% of patients experiencing complete or partial symptomatic recovery under standard medical management [23]. This high rate of clinical recovery is mirrored structurally, with complete vessel recanalization occurring in approximately 60% of cases [24].

## Conclusions

We describe a rare case of Villaret's syndrome manifesting with multiple CN palsies and Horner’s syndrome resulting from ICAD following exertion, and presenting in the absence of intracranial ischemia. It highlights the importance of recognizing the constellation of clinical findings in this syndrome, which should not be mistaken for an acute stroke, to ensure appropriate diagnostic investigations are promptly initiated. A vascular etiology of Villaret's syndrome is rare, but the acute, rapid onset was significant; a high index of clinical suspicion prompted an MRI, which successfully identified the underlying dissection when initial CTA was unrevealing.

Overall, the prognosis for recovery from ICAD-related local complications remains highly favorable, and our patient's symptoms completely resolved following standard first-line antithrombotic therapy.
